# Respirable Aerosol Production and Reduction of Avian Influenza Transmission Risk during Chicken Processing, Bangladesh 

**DOI:** 10.3201/eid3204.251878

**Published:** 2026-04

**Authors:** Nadia Ali Rimi, Md. Khaled Saifullah, Md. Habibullah Fahad, Kamal Hossain, Rebeca Sultana, Ireen Sultana Shanta, David E. Swayne, Syed Mohammad Golam Mortaza, Md. Giasuddin, Md. Zakir Hassan, Christopher LeBoa, Debashish Biswas, Mahbubur Rahman, Joshua A. Mott, Erin D. Kennedy, William G. Lindsley

**Affiliations:** University of Glasgow, Glasgow, Scotland, UK (N.A. Rimi); icddr,b, Dhaka, Bangladesh (N.A. Rimi, M.K. Saifullah, M.H. Fahad, K. Hossain, R. Sultana, I.S. Shanta, S.M.G. Mortaza, D. Biswas); University of Georgia, Athens, Georgia, USA (D.E. Swayne); Agricultural Research Service, US Department of Agriculture, Athens (D.E. Swayne); Bangladesh Livestock Research Institute, Dhaka (M. Giasuddin, M.Z. Hassan); University of California, Berkeley, California, USA (C. LeBoa); The University of Western Australia, Crawley, Western Australia, Australia (D. Biswas); Institute of Epidemiology, Disease Control and Research, Directorate General of Health Services, Dhaka (M. Rahman); Centers for Disease Control and Prevention, Atlanta, Georgia, USA (J.A. Mott, E.D. Kennedy); Centers for Disease Control and Prevention, Morgantown, West Virginia, USA (W.G. Lindsley)

**Keywords:** influenza, viruses, respiratory infections, zoonoses, avian influenza viruses, particulate matter, airborne transmission, live bird market, Bangladesh

## Abstract

In Bangladesh, influenza A(H5N1) viruses are endemic in poultry. Processing infected chickens can aerosolize viruses, increasing the risk for human infections. We evaluated particulate matter (PM_2.5_) mass concentration during slaughtering and defeathering methods used in live bird markets in Bangladesh to identify solutions to reduce aerosol exposure. We slaughtered 675 chickens using cones and barrels with 3 lid types and defeathered 45 chickens using a defeathering machine with 5 lid types. We interviewed 3 slaughterers to understand method preference. For slaughtering, barrels with a solid or star-cut lid reduced PM_2.5_ mass concentrations by 65%–73% compared with uncovered barrels. For defeathering, machines fully covered by a solid lid or lid with a hole and pivot door reduced PM_2.5_ mass concentrations by 50% compared with machines with no lid. Slaughterers preferred barrels covered with solid lids and defeathering machines covered with solid or hinged lids. Those methods might reduce aerosol exposure during poultry processing.

The possible human-to-human transmission of respiratory pathogens such as novel influenza viruses and coronaviruses poses an ongoing pandemic risk (*1*,*2*). Highly pathogenic avian influenza (HPAI) A(H5N1) viruses pose a major concern because of the potential for animal-to-human transmission and severe outcomes in humans (*3*). In addition, influenza A viruses, including H5N1, have pandemic potential because of their ability to reassort and mutate, possibly leading to novel strains that can spread efficiently among humans (*4*). As of November 2025, a total of 992 human cases of influenza A(H5N1), including 476 deaths (48% case-fatality rate), had been reported across 25 countries (*5*).

Live bird markets (LBMs) are globally recognized as high-risk environments for the transmission and possible reassortment of avian influenza viruses (AIV) (*6*–*8*), including in Bangladesh, where HPAI H5N1 is endemic in poultry (*9*). Transmission of AIV through aerosols is of concern because of the potential for respirable aerosols containing particulate matter <4 µm to penetrate deeply into the lungs during inhalation (*10*). Human exposure to the slaughtering and processing of sick or healthy-appearing poultry in LBMs has frequently been identified as a cause of AIV infections (*7*,*11*–*14*), and the detection of AIV RNA in environmental samples and workers underscores the persistent risk for transmission (*15*–*17*).

The slaughtering practices in LBMs are often conducted without adequate safety measures (*18*) and can contribute to generating and disseminating aerosolized particles into the human breathing zone, particularly during exsanguination and mechanical defeathering of slaughtered poultry (*19*). Recognizing the need for context-specific interventions, recent efforts have focused on evaluating methods to reduce aerosol production during poultry slaughtering and defeathering (*19*–*21*). We measured particulate matter mass concentration, a commonly used measure of indoor air pollution consisting of the total mass concentration of all airborne particles <2.5 microns in diameter. In this study, we used particulate matter 2.5 (PM_2.5_), indicating particulates that are <2.5 μm in diameter, as a proxy for aerosolized virus to identify methods that mitigate aerosol production during exsanguination and mechanical defeathering of slaughtered chickens.

## Materials and Methods

### Study Site

During January–March 2020, we replicated the slaughtering and defeathering steps commonly performed in LBMs and employed commonly used tools within a constructed booth in the National Reference Laboratory for Avian Influenza, Bangladesh Livestock Research Institute (BLRI), Savar, Dhaka (Appendix Table) (*19*). To conduct the experiments, the team followed similar procedures to those described in the pilot experiment (*19*).

### Animal Selection

We used broiler chickens (*Gallus gallus domesticus*), a commonly sold species in Bangladesh LBMs (*22*). All chickens were normal market weight (≈1.7 kg) (*19*) and appeared healthy. A total of 750 chickens were used in the study: 30 chickens were used to test and standardize each experimental condition (1–2 chickens per condition), 675 were used for slaughtering experiments, and 45 were used for defeathering experiments.

### Placement of Aerosol Particle Monitors

**Figure 1:**
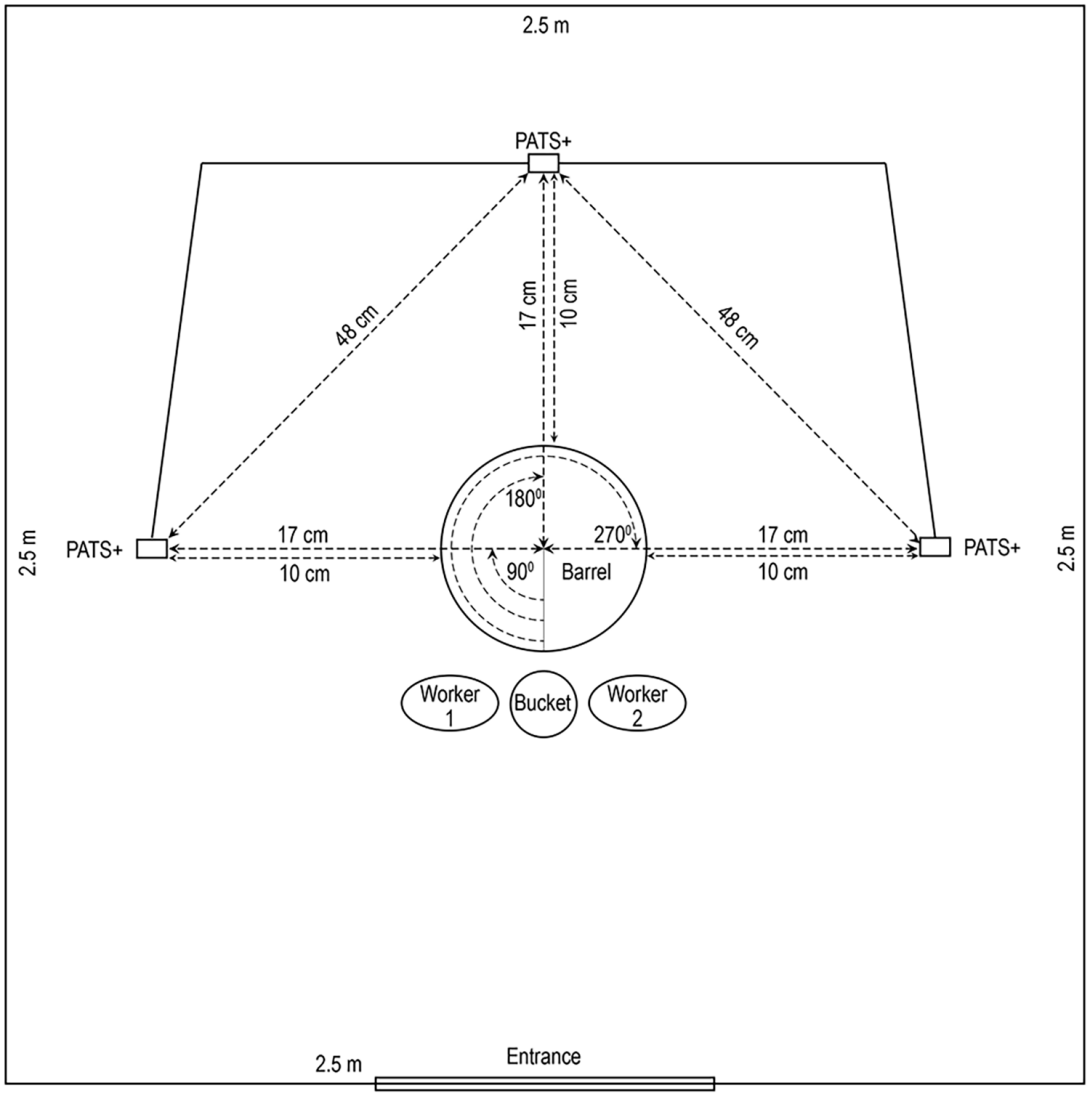
Diagram of placement of equipment and particle monitors inside booth for chicken slaughtering experiments at Bangladesh Livestock Research Institute, Savar, Dhaka, 2020, in study of respirable aerosol production and reduction of avian influenza transmission risk during chicken processing. The PATS+ monitors (Berkeley Air Monitoring Group, https://berkeleyair.com) were positioned at human breathing level (148 cm) in 3 angular positions: left (90°), opposite (180°), and right (270°) relative to the entrance. PATS+, Particle and Temperature Sensor Plus.

**Figure 2:**
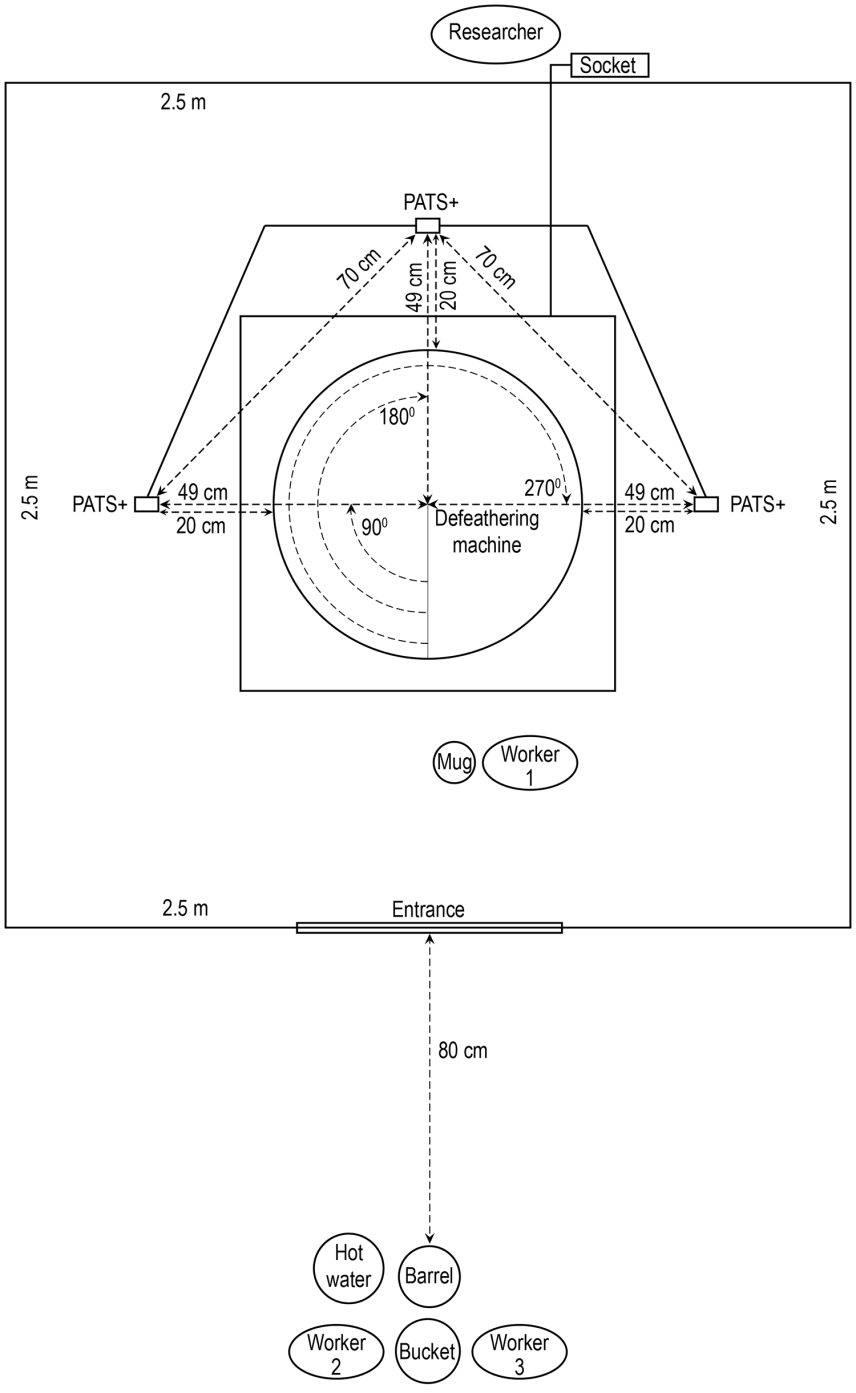
Diagram of placement of equipment and particle monitors inside and outside booth for chicken defeathering experiments at Bangladesh Livestock Research Institute, Savar, Dhaka, 2020, in study of respirable aerosol production and reduction of avian influenza transmission risk during chicken processing. The PATS+ monitors (Berkeley Air Monitoring Group, https://berkeleyair.com) were positioned at human breathing level (148 cm) in 3 angular positions: left (90°), opposite (180°), and right (270°) relative to the entrance. PATS+, Particle and Temperature Sensor Plus.

We used Particle and Temperature Sensor Plus (PATS+) particle monitors (Berkeley Air Monitoring Group, https://berkeleyair.com) to assess aerosolized PM_2.5_ mass concentration (µg/m^3^) during slaughtering and defeathering (*23*) (Appendix Table). To optimize measurement of PM_2.5_ mass concentrations without interfering with slaughtering (Figure 1) and defeathering (Figure 2) processes, we placed the monitors 17 cm from the barrel or defeathering machine and positioned at human breathing level (148 cm) (*19*) in 3 directions: left (90°), opposite (180°), and right side (270°) relative to the entrance (Figures 1, 2).

### Experiment Setup and Procedures

We hired 3 workers from the nearest local LBM on the basis of their experience in slaughtering and defeathering with commonly used equipment and their willingness to work with PPE inside the booth at BLRI. We trained each worker in the study procedure and rotated roles during the experiment to ensure consistency and minimize variability in results. We recorded baseline measurements of aerosol particles inside the booth for 5 minutes before initiating each experiment, when no slaughtering or defeathering activity was ongoing. The researchers recorded the timing of each slaughtering and defeathering and the temperature and humidity during each experiment.

### Slaughtering Experiments

**Figure 3:**
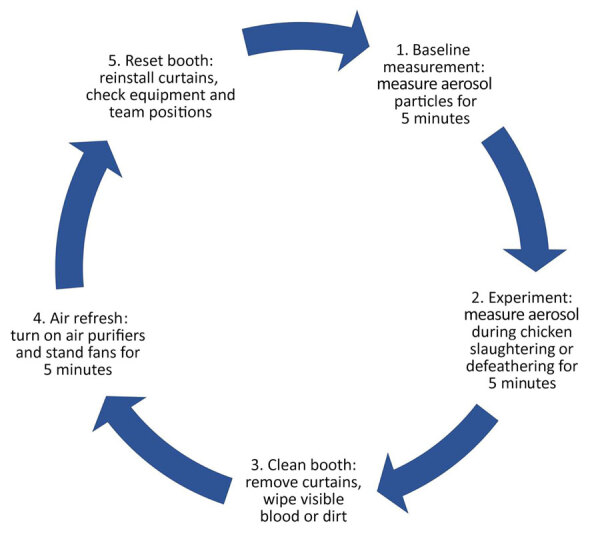
Chronology of each experimental event in study of respirable aerosol production and reduction of avian influenza transmission risk during chicken processing, Bangladesh Livestock Research Institute, Savar, Dhaka, 2020. Events were repeated consistently to ensure uniformity throughout the experiments. Baseline measurement of particulate matter <2.5 μm in diameter mass concentrations were measured for 5 minutes before each experiment. Aerosol measurements during processing included single chicken slaughtering (1 chicken at a time), multiple chicken slaughtering (4 chickens at a time), and single chicken defeathering (1 chicken at a time).

We used plastic barrels or slaughtering cones to contain chickens during exsanguination (Appendix Table). We used 2 types of lids to cover the plastic barrel: a solid lid and a star-cut lid (Appendix Figure 1) (*19*). We conducted 2 types of slaughtering experiments: single chicken slaughter (1 chicken per experiment) and multiple chicken slaughter (4 chickens per experiment). We performed each slaughter across 5 intervention types: open barrel without a lid, barrel covered with a solid lid, barrel covered with a star-cut lid, small cone, and large cone (Appendix Figure 1). Inside the booth, 2 workers slaughtered a chicken with a knife over the bucket and immediately put it inside the barrel or cone for its exsanguination. Open barrel experiments used no lid. For covered barrel experiments, barrels were covered with solid or star-cut lids. During multiple-slaughter experiments, chickens were quickly put inside the barrel one after another after slaughtering by sliding the solid lid or by inserting them through the star cut on the lid. We recorded aerosol PM_2.5_ mass concentration measurements from the point the worker entered the booth until 5 minutes afterwards. A worker then removed the chicken from the barrel or cone and the booth. We recorded the death struggle time of each slaughtered chicken during the experiments, defined as the time between placement of the chicken into the barrel or cone after slaughtering and the cessation of visible body movements or sound or movement of flapping of the chicken from inside the barrel. In the case of multiple-chicken slaughtering, we considered the death struggle time as the time between placement of the first slaughtered chicken into the barrel or cone and cessation of movement or sound of the last chicken. We refreshed the room and booth air between each slaughtering event by removing the booth curtains and turning on air purifiers and stand fans for 5 minutes. We then recorded a baseline measurement for 5 minutes. The door and windows of the room were closed throughout the experiment. A previous study showed that aerosol PM_2.5_ mass concentrations peaked and then fell within a 5-minute interval during poultry slaughtering events as recorded with the PATS+; concentrations returned close to the baseline after 5 minutes of refreshing time (*19*). We repeated the procedure 27 times for each intervention type (Figure 3).

### Defeathering Experiments

The defeathering experiments consisted of 5 defeathering machine lid modifications: open machine without a lid, machine half-covered by a hinged lid (to allow for pouring water) that could be closed fully if needed, machine partially covered by a lid with a hole smaller than the defeathering machine’s mouth (*19*,*21*), machine fully covered by a lid with a hole and pivot door (to cover the hole once the chicken was placed inside), and machine fully covered by a solid lid (Appendix Figure 2). Inside the booth, we placed a defeathering machine and a bucket of room-temperature water on the ground in the middle of the booth. Outside the booth, we set up a bucket, a barrel, and a container with hot water (60°C) 80 cm away from the booth. Two workers slaughtered a chicken outside the booth with a knife over the bucket, then placed the chicken inside the barrel and covered the barrel for exsanguinations for 160 seconds. The slaughterer dipped the carcass in hot water for 17 seconds, carried the chicken inside the booth using a bucket, placed it inside the defeathering machine, and ran the machine for 20 seconds. While the machine was running, the worker poured 2.5 L of room-temperature water into the machine by moving the solid lid, pouring through the hole or uncovered half of the hinged lid, or moving the pivot door of the lids. No lid was used for open defeather experiments. For covered defeather experiments, the machine was fully covered with solid lid or partially covered with the hinged or customized lids. We recorded aerosol PM_2.5_ mass concentration measurements for 5 minutes from the point the worker entered the booth. We repeated the experiment 9 times for each lid modification. We performed the same procedure of taking a baseline measurement and refreshing the air in the booth before each defeathering experiment, as was performed for the slaughtering experiments (Figure 3).

### Sample Size Calculation

A previous pilot study reported an average PM_2.5_ mass concentration of 75.8 µg/m^3^ (SD 55.5 µg/m^3^) during chicken slaughtering in an open barrel with the PATS+ (*24*). On the basis of that finding, we assumed a minimum difference in the mean of 50% (37.9 µg/m^3^) compared with the open barrel mean of 75.8 µg/m^3^; we assumed a common SD of 55.5 µg/m^3^. Using a 1-sided t-test with a 5% significance level and 80% power, the estimated sample size was 135 poultry for single slaughter and 540 poultry for multiple slaughter experiments (27 per arm). Similarly, the previous experiment also found an average PM_2.5_ mass concentration of 19.9 µg/m^3^ (SD 8.2 µg/m^3^) during defeathering using a defeathering machine without a lid (*24*). Assuming a minimum difference in the mean of 50% (9.9 µg/m^3^) from the open defeathering mean of 19.9 µg/m^3^ and using a 1-sided t-test with a 5% significance level and 80% power, the estimated sample size was determined to be 45 poultry (9 per arm).

### Qualitative Data Collection

We conducted an in-depth interview with each of the 3 workers who had been employed to conduct the experiment to understand the advantages, disadvantages, feasibility, durability, and likelihood of adoption of each method (Appendix). Because of COVID-19 lockdown restrictions imposed by the government of Bangladesh, those interviews were conducted over mobile phone within 1 month of completing the experiments to minimize recall bias.

### Data Analysis

We summarized the PM_2.5_ mass concentrations using the mean and SD for each slaughtering and defeathering method, on the basis of the recorded concentrations from all experiments conducted under each condition combined and across different placements of the particle monitor. The open barrel and defeathering machine without a lid were used as reference for comparison among the different methods, because these practices were common (*19*) and were assumed to produce the highest mass concentrations. A 2-sample t-test was used to compare mean mass concentrations, and we reported the percentage difference of mean PM_2.5_ mass concentration with 95% CI for each slaughtering and defeathering method, using Poisson regression with robust variance. Because we used the baseline data for each type of experiment (single and multiple slaughtering and defeathering) to test 4 different hypotheses, we considered 1.25% as level of significance instead of 5% according to Bonferroni correction to account for multiple comparisons. We conducted data management and analysis using Stata software version 15 (StataCorp, LLC, https://www.stata.com) and performed data visualization using R version 4.3.2 (The R Project for Statistical Computing, https://www.r-project.org).

We transcribed interviews verbatim and analyzed them manually using inductive coding to identify emerging themes. Two authors independently read through the transcripts and collaboratively developed initial codes. Those codes were refined through discussion, leading to identifying themes that captured key insights. We then summarized coded data according to the study objectives and relevant themes.

### Ethics

This study was reviewed and approved by the Institutional Review Boards of icddr,b (protocol number PR-19053) and US Centers for Disease Control and Prevention (see 45 C.F.R. part 46 and 21 C.F.R. part 56 and Protocol number 7237) and Institutional Animal Care and Use Committee (protocol number 3054KILCHIX). All participants provided written consent.

## Results

**Figure 4:**
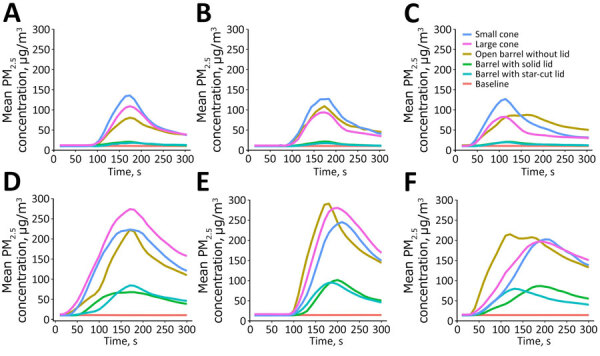
Average PM_2.5_ mass concentration during single and multiple chicken slaughtering methods in study of respirable aerosol production and reduction of avian influenza transmission risk during chicken processing, Bangladesh Livestock Research Institute, Savar, Dhaka, 2020. Results reported by Particle and Temperature Sensor Plus monitors (Berkeley Air Monitoring Group, https://berkeleyair.com) are shown for each angle position of sensor. A) Single chicken slaughtering; angle 90°. B) Single chicken slaughtering; angle 180°. C) Single chicken slaughtering; angle 270°. D) Multiple chicken slaughtering; angle 90°. E) Multiple chickens slaughtering; angle 180°. F) Multiple chicken slaughtering; angle 270°. PM_2.5_, particulate matter <2.5 μm in diameter.

**Figure 5:**
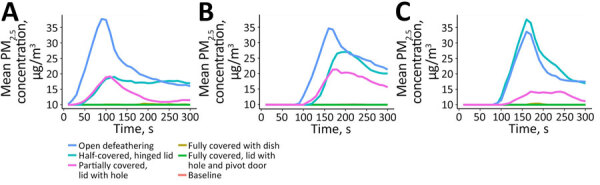
Average PM_2.5_ mass concentration during single chicken defeathering in study of respirable aerosol production and reduction of avian influenza transmission risk during chicken processing, Bangladesh Livestock Research Institute, Savar, Dhaka, 2020. Results reported by Particle and Temperature Sensor Plus monitors (Berkeley Air Monitoring Group, https://berkeleyair.com) are shown for angle position of sensor and time of measurement in seconds: A) 90° angle; B) 180° angle; C) 270° angle. PM_2.5_, particulate matter <2.5 μm in diameter.

The average PM_2.5_ mass concentrations during baseline for all experiments were relatively consistent, ranging from 10.0 to 12.6 µg/m^3^ (Table; Figures 4, 5), which is near the limit of detection (LOD) of the particle monitors used (LOD = 10 µg/m^3^) (Appendix Table). Higher variations in baseline PM_2.5_ mass concentrations (range 10.01–36 µg/m^3^) were observed in 109 of 810 total readings from 3 monitors during 270 slaughter experiments.

**Table:** PM_2.5_ mass concentration across combined sensor positions during experiments of chicken slaughtering and defeathering methods in study of respirable aerosol production and reduction of avian influenza transmission risk during chicken processing, Bangladesh Livestock Research Institute, Savar, Dhaka, 2020*

Method	Mean PM_2.5_ mass concentration during baseline, µg/m^3 ^(min–max)†	Mean PM_2.5_, µg/m^3^ (SD)	PM_2.5_ mass concentration difference from referent, µg/m^3^	Percentage of reduced PM_2.5_ mass concentration (95% CI)	p value‡
Single chicken slaughtering, n = 27					
Open barrel without lid	11.1 (10–18)	49.6 (25.4)	Referent	–	–
Barrel covered with solid lid	10.1 (10–12)	14.1 (5.5)	–35.5	–71.6 (–77.7 to –63.9)	<0.001
Barrel covered with star-cut lid	10 (10–10)	13.3 (5.2)	–36.2	–73.1 (–78.9 to –65.8)	<0.001
Small cone	10.3 (10–16)	57.4 (47.7)	7.8	15.7 (–19.6 to 66.6)	0.458
Large cone	11.4 (10–24)	44.4 (31.6)	–5.2	–10.5 (–35.5 to 24.2)	0.509
Multiple chicken slaughtering, n = 27§					
Open barrel without lid	10.4 (10–14)	130.1 (27.5)	Referent	–	–
Barrel covered with solid lid	10 (10–10)	36 (13.6)	–84.5	–64.8 (–69.2 to –59.7)	<0.001
Barrel covered with star-cut lid	10.7 (10–20)	34.3 (21.2)	–85.5	–65.5 (–71.9 to –57.7)	<0.001
Small cone	10.2 (10–12)	112 (52.3)	–8.3	–6.3 (–21.6 to 11.9)	0.470
Large cone	12.6 (10–36)	131 (60.1)	13.1	10 (–8.2 to 31.8)	0.327
Single chicken defeathering, n = 9					
Open machine without lid	10 (10–10)	20 (6.5)	Referent	–	–
Machine half covered by hinged lid	10 (10–10)	17.5 (6.7)	–2.5	–12.5 (–36.4 to 20.5)	0.438
Machine partially covered by lid with hole	10 (10–10)	13.3 (3.4)	–6.7	–33.5 (–49.0 to –13.3)	0.019
Machine fully covered by lid with hole and pivot door	10 (10–10)	10.0 (0.0)	–10	–50 (–59.4 to –38.5)	0.002
Machine fully covered by solid lid	10 (10–10)	10.0 (0.1)	–10	–49.9 (–59.3 to –38.3)	0.002

*PM_2.5_, particulate matter <2.5 μm in diameter. †PM_2.5_ mass concentrations were measured for 5 minutes before each experiment. ‡Calculated using 2-sample t-test. 1.25% was considered as level of significance instead of 5% according to Bonferroni correction. §Slaughtering events with 4 chickens at a time.

PM_2.5_ mass concentrations varied across different slaughtering methods compared with the open barrel experiments, where the mass concentration was 49.6 µg/m^3^ for single slaughtering and 130.1 µg/m^3^ for multiple slaughtering (Table; Figure 4). In both single and multiple slaughtering experiments, covering the barrel with a solid lid significantly reduced PM_2.5_ mass concentrations. For single slaughtering, the reduction was 71.6% (95% CI 63.9%–77.7%), whereas for multiple slaughtering, the reduction was 65% (95% CI 59.7%–69.2%). We observed a similar reduction trend for barrels covered with a star-cut lid: 73.1% (95% CI 65.8%–78.9%) for single slaughtering and 65.5% (95% CI 57.7%–71.9%) for multiple slaughtering. The use of slaughtering cones did not significantly reduce aerosol levels for any slaughtering experiments (Table; Figure 4). During defeathering experiments, fully covering the machine with a solid lid or a lid with a hole and pivot door reduced PM_2.5_ mass concentrations to minimum detectable levels of 10 µg/m^3^. The reduction in both cases was 50% (95% CI 59.3%–38.3%) (Table; Figure 5).

**Figure 6:**
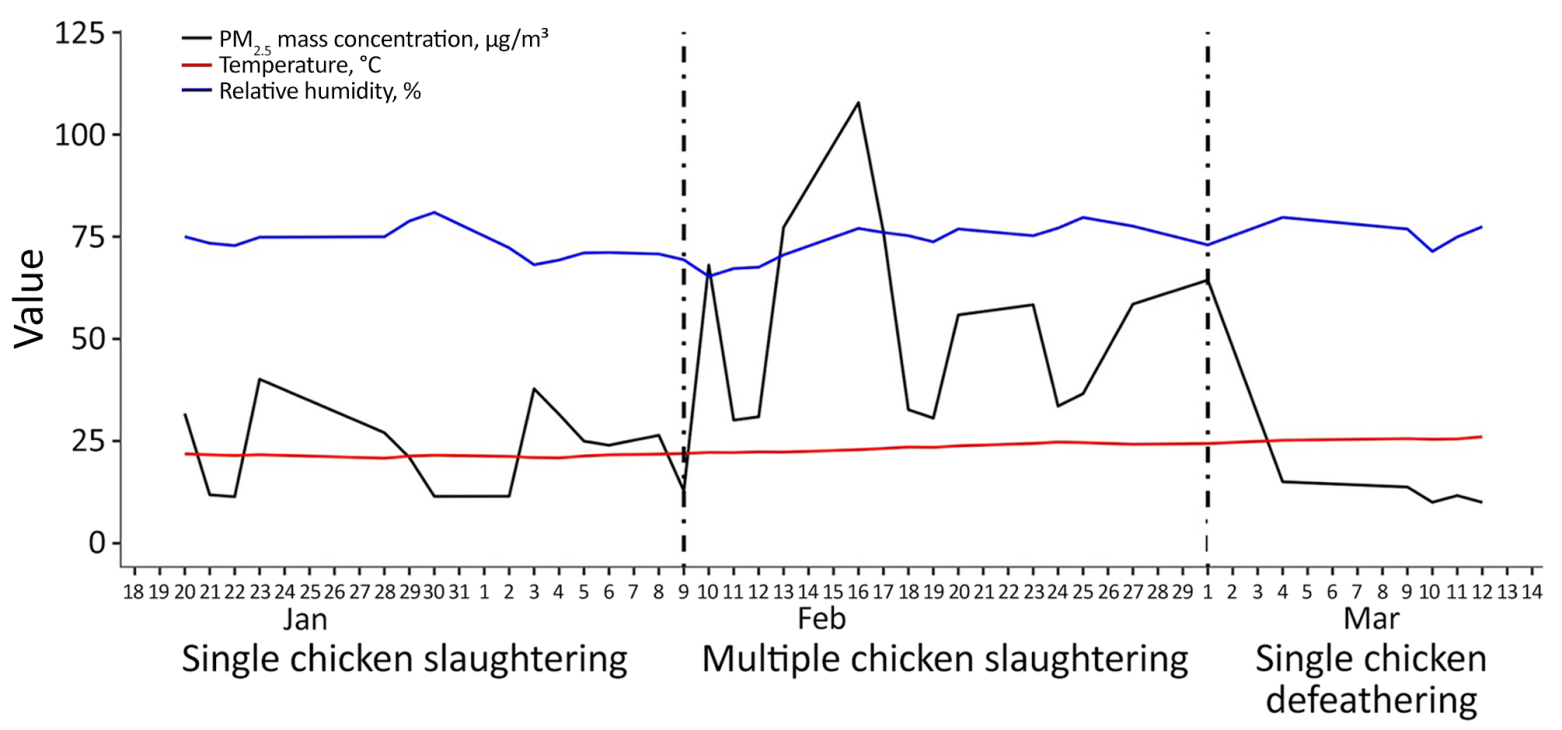
Trend and variability of average particulate matter mass concentration, temperature, and relative humidity in study of respirable aerosol production and reduction of avian influenza transmission risk during chicken processing, Bangladesh Livestock Research Institute, Savar, Dhaka, 2020. Results are shown for single (1 chicken at a time) and multiple (4 chickens at a time) chicken slaughtering events and defeathering events (1 chicken at a time). PM_2.5_, particulate matter <2.5 μm in diameter.

The relative humidity fluctuated slightly but remained generally stable (mean 53%, SD 5.1%), and temperature was also maintained without substantial variation throughout the experiment period (mean 25°C, SD 1.4°C) (Figure 6). The average duration of the death struggle was 97 (range 94–101) seconds for single slaughtering experiments and 122 (range 112–134) seconds for multiple slaughtering experiments.

The average age of the 3 hired workers was 20 (range 19–21) years; the average amount of experience work in the LBMs was 7.5 years, and all had completed primary education. Workers preferred the barrel covered with a solid lid method for poultry slaughtering, followed by the barrel covered with a star-cut lid, citing those tools’ usefulness in preventing blood splattering and their ease of operation (Appendix). However, methods involving cones were met with less enthusiasm because of their unsuitability for containing >4–6 poultry at a time during busy hours and their higher associated costs (Appendix). For defeathering, workers expressed a strong preference for full cover with a solid lid, citing their familiarity with its operation and ease of use (Appendix). The half-covered hinged lid, which enabled water pouring during the process, also garnered favor among workers for its practicality. Workers were less inclined toward the lid with a hole and pivot door method, because it was less familiar and was perceived as more challenging to use (Appendix).

## Discussion

Our findings show that PM_2.5_ mass concentrations increased during chicken slaughtering and defeathering events across all experimental conditions; each method influenced aerosolized mass concentration levels in varying ways. Covering slaughtering containers with solid or star-cut lids and fully covering defeathering machines consistently resulted in substantial reductions in aerosolized PM_2.5_ mass concentrations. Those methods were also preferred by the workers.

The open barrel method, identified as the most commonly used slaughtering practices in Dhaka City (*19*), increased airborne PM_2.5_ by 38.5 μg/m^3^ for a single chicken slaughter and 120.1 μg/m^3^ for multiple chicken slaughter over the baseline PM_2.5_ mass concentrations measured before any slaughtering took place. During both single and multiple slaughtering experiments, barrels covered with either a solid lid or a star-cut lid showed the greatest reductions in PM_2.5_ mass concentrations compared with open barrels. Multiple slaughtering produced a higher mass concentration of PM_2.5_, likely because of the cumulative aerosol production from multiple chickens. Cone-based slaughtering methods increased aerosolization more than the open barrel. That outcome could be because of the distance between the slaughtered chicken and the monitor was relatively short in cones, where the chickens were positioned ≈50 cm above ground instead of at ground level (Appendix Table). Those findings suggest that improved slaughtering methods, such as the use of covered barrels, could reduce infectious disease exposure and the circulation of airborne pathogens in LBMs, thereby mitigating the risk for zoonotic transmission.

The fully covered defeathering machines (e.g., those with a solid lid or a lid with a hole and pivot door) reduced PM_2.5_ mass concentrations to the minimum detectable levels of 10 µg/m^3^, demonstrating their ability to prevent aerosol escape. Defeathering machines that were partially covered (e.g., with hinged lids or lids with holes) generated detectable levels of aerosol PM_2.5_ mass concentration, although those levels were lower than those observed with completely open defeathering machines. That finding suggests that even partial coverings can reduce aerosol concentrations but that they are less effective than fully closed methods. A survey conducted in LBMs of Dhaka City in 2018 found that 33% of shops used mechanical defeathering (*19*), which substantially increased the total number of generated aerosol PM_2.5_ mass concentrations over manual defeathering (*20*). The use of such motor-driven devices produces strong air circulation, which is favorable for generating and dispersing aerosolized particles and is discouraged (*20*). However, such devices are increasingly popular, particularly for defeathering broiler chickens in cities (*19*). Fully covering the defeathering machine with lids might play a role in preventing aerosolized particles from escaping, potentially reducing the risk for airborne transmission of pathogens such as HPAI H5N1.

Some countries have banned LBMs to reduce risk for AIV spread (*25*,*26*). In a Muslim-majority country such as Bangladesh, adherence to halal dietary laws while slaughtering poultry requires using a well-sharpened knife to make a swift, deep incision that cuts the front of the esophagus, trachea, the jugular veins, and the carotid arteries; lining up the head of the animal to be slaughtered in the direction of the Mecca; and pronouncing Islamic invocation (*27*,*28*). A common cultural preference of consumers in Muslim-majority countries is to observe the slaughter to ensure halal requirements are met and also to verify the health and quality of the poultry (*29*). For that reason, poultry is mostly traded at LBMs (*30*), where slaughtering of live birds is commonly practiced, and efforts to ban or control markets are less likely to succeed. Therefore, improving the environment to limit the spread of viruses in LBMs is crucial. Using covered barrels and fully closed defeathering machines could be simple yet effective methods to reduce aerosol production in LBMs, where implementing more advanced biosecurity measures might be challenging because of infrastructural or economic constraints. Interventions that can involve personal discomfort, such as use of masks, are often resisted by market workers, as was observed in a behavior change intervention that identified discomfort as a reason for not following recommendations to cover the nose and mouth during poultry slaughtering (*31*). Using covered barrels and defeathering machines requires minimal behavioral change and can be integrated seamlessly into existing workflows, increasing the likelihood of adoption and sustained use. The practices of LBMs in Bangladesh, such as exsanguination into barrels or cones and mechanical defeathering, are not unique to Bangladesh; they are also used in countries such as Pakistan (*32*), Burkina Faso (*33*), and China (*34*). Our findings might be relevant for countries with LBMs and similar slaughtering practices, particularly in resource-limited Muslim communities and other settings where on-site poultry processing remains common.

The first limitation of our study is that it focused on measuring aerosol concentrations as a proxy for potential viral transmission risk and did not directly assess the presence or infectivity of AIV in the aerosols produced. We assume that aerosolized PM_2.5_ mass concentration is proxy for AIV in this setting because AIVs are prevalent in the air of LBMs (*35*). However, the use of PM_2.5_ as a proxy for AIV or other bioaerosols is not well understood and does not reliably estimate infectious dose or AIV particles generated in the environment. The exclusive focus on PM_2.5_ in this study might restrict a comprehensive understanding of aerosol size distribution relevant to bioaerosol transmission. Future studies could include other size fractions, such as PM_1_ and PM_10_, to provide a more complete characterization of aerosolized particles involved in AIV transmission. Second, the experiments were conducted in a controlled environment with a specific poultry species, which might not fully replicate the conditions of LBMs, where factors such as airflow, crowd density, variety in poultry species (including duck), and variability in worker practices could influence aerosol dynamics and AIV infection risk. Despite those limitations, this study provides a critical roadmap for future experiments by establishing baseline data for aerosol generation during slaughtering and defeathering methods.

In conclusion, this study provides evidence that specific containment methods during exsanguination and mechanical defeathering of slaughtered chickens can substantially reduce aerosolized PM_2.5_ mass concentration, potentially mitigating the risk for AIV transmission in LBMs to poultry workers and customers. Implementing those measures could be an effective, feasible, and acceptable strategy to enhance biosecurity in settings where AIV poses a persistent threat to both poultry and human health. Future studies could incorporate virological assessments to better understand the relationship between aerosol concentrations and actual viral transmission risk. Integrating lid-based interventions with other biosecurity measures, such as improved ventilation, regular disinfection, and worker health monitoring, might provide a more robust defense against zoonotic transmission of the virus. 

Appendix Additional information about respirable aerosol production and reduction of avian influenza transmission risk during chicken processing, Bangladesh.
