# Supplementary material for: Respirable Aerosol Production and Reduction of Avian Influenza Transmission Risk during Chicken Processing, Bangladesh
Source: Emerg Infect Dis. 2026 Apr;32(4):603–13. doi: 10.3201/eid3204.251878 (PMC13094850; doi:10.3201/eid3204.251878)
Supplement: Appendix — Additional information about respirable aerosol production and reduction of avian influenza transmission risk during chicken processing, Bangladesh. [file 25-1878-Techapp-s1.pdf]

Article DOI: https://doi.org/10.3201/eid3204.251878

EID cannot ensure accessibility for supplementary materials supplied by authors. Readers who have difficulty accessing supplementary content should contact the authors for assistance.

Respirable Aerosol Production and Reduction of Avian Influenza Transmission Risk during Chicken Processing, Bangladesh

Appendix

Topic Guide 1: Themes explored during in-depth interview with slaughterers of the experiment

Background Information: Age, educational qualifications, work experience, working hours

For slaughtering method

• Explore the advantages and disadvantages of using different slaughtering methods (e.g., open barrel, closed barrel, closed barrel with star-cut lid, small slaughtering cone and large slaughtering cone) for both single or multiple chickens at a time.

• Explore their familiarity, feasibility and user-friendliness of each method, i.e., which method is easy or possible to follow, and which are not (explore each method individually), and the availability and estimated cost in local market of the equipment that are used in each method, e.g., barrel, solid lid, star-cut lid, small and large slaughtering cones. Explore reasons behind each response.

• Explore which method they would prefer or not prefer using and why or why not (explore each method individually).

For defeathering method

• Explore the advantages and disadvantages of using different defeathering methods (e.g., open defeathering/ fully closed defeathering/ half-closed defeathering).

• Explore their familiarity, feasibility and user-friendliness of each method, i.e., which method is easy or possible to follow, and which are not (explore each method individually), and the availability and estimated cost in local market of the equipment that are used in each method, e.g., hinged lid, solid lid, lid with a hole, and lid with a hole and pivot door. Explore reasons behind each response.

• Explore which method they would prefer or not prefer using and why or why not (explore each method individually).

Worker feedback on methods explored in study:

Workers' opinions about advantages, disadvantages, and feasibility of the methods

a. Workers' preference for barrels considering usefulness:

"Slaughtering is difficult with small cones. If the poultry are large, the chickens jump and splatter blood. When using a barrel, it's better to have a lid. If I put the lid back on, the blood doesn't splatter on my body."

b. Workers' preference for barrels considering cost:

"Cones are more expensive than barrel. The barrel we use for slaughtering costs around US$ 4, while a cone costs around US$ 10. If the cone is made from heavy, high-quality material, the price will be higher. If it's made from a lighter tin sheet, it will erode quickly."

c. Workers' preference for solid lids:

"We mostly use hinged lids or dishes (i.e., solid lids), particularly hinged lid. When it (hinged lid) gets damaged, people switch to using a dish as a lid. The hinged lid isn't replaced because it spoils at the joints, whereas the dish lasts longer. Covering with a dish doesn't let blood or water to spill. The dish serves multiple purposes — you can place giblets on it, or even other items of the shop."

d. Workers' disfavoring the lid with a hole and pivot door:

"It will be difficult to lift the chicken with the new lids (lid with a hole and pivot door). Although easy to clean, the mantling and dismantling daily is a problem. It is difficult to pick up

the chicken after defeathering. The hole in the middle is small. It is difficult to pour water. If water is not poured appropriately, the chicken will not be clean."

Appendix Table. Equipment used for chicken slaughtering and defeathering experiments at Bangladesh Livestock Research Institute (BLRI), Savar, Dhaka, 2020

Equipment	Description and dimensions	Purpose
For all experiments		
Booth	Square shaped (2·5m width x 2·5m length x 2m height) made with 13 mm PVC plastic pipe and transparent cellulose films	To minimize extraneous air movement and aerosol particle dispersion during experiments
Stand fans	Two stand fans (diameter: 24 inches; height: 60 inches; rotations per minute (RPM): 1250)	To refresh the booth air between experiments to keep the baseline $PM_{2.5}$ mass concentration consistent throughout all experiments
Air purifier	Two Hitachi air purifiers with HEPA filters (model: EP-A6000)	
PPE	Non-sterile latex gloves, goggles, apron, rubber boots and N95 respirators	Used by the workers and researchers for their protection during experiments
PATS+ particle monitors	A portable aerosol monitor (developed by Berkeley Air Monitoring Group, Berkeley, CA, USA) that measures the concentration of aerosol particles ≤2·5 μm using optical scattering sensor. The instrument has a precision of±15%. The monitor takes count of particulate matter ($PM_{2.5}$) mass concentration in every 10 s throughout the duration. Its lower detection limit for $PM_{2.5}$ is 10–20 μg/m^3, and the upper limit is 30,000–50,000 μg/m^3 (*1*).	To assess aerosolized respirable particles levels during slaughtering and defeathering experiments
Iron frame	Placed to surround the barrel, cone and defeathering machines on three sides; height: 144 cm	To hold the particle monitors
Knife	Locally available; made of metal	To slaughter chickens
Barrel	Plastic; diameter: 25·4 cm; height: 55·9 cm	Contain the slaughtered chicken during exsanguination
Plastic bucket	Diameter: 26·7 cm; height: 25·4 cm	For blood collection
Mosquito coil	Locally available; usually made into a spiral, and typically made using dried paste of pyrethrum powder	To use for the smoke test to confirm the absence of air movement inside the booth
For slaughtering experiments		
Barrel lids	Solid plastic lid: diameter 28 cm<br>Star-cut plastic lid: diameter 28 cm	To cover the barrel and to allow insertion of chickens into barrel<br>- by sliding the lid (solid lid)<br>- through star-cut that facilitated putting slaughtered chickens inside the barrel without removing or sliding the lid (star-cut lid)
Slaughtering cones	Small cone (per cone): Radius: 17 cm; depth: 32 cm; height: 50 cm<br>Large cone (per cone): Radius: 20 cm; depth: 34 cm; height: 50 cm	To contain the chicken during exsanguination
Bricks	Length: 23 cm; height: 9 cm, weight: ~2·5 kg	Two pieces placed inside the barrel and on the cone tray to stabilize the barrel and cones during exsanguination of the slaughtered chickens
For defeathering experiments		
All slaughtering equipment	As described above	As described above
Defeathering machine	A square box: length: 68·6 cm; width: 68·6 cm; height: 57·2 cm<br>A cylinder: diameter: 58·4 cm; height: 49·5 cm; Total height: 110 cm	To remove feathers
Lids for covering the defeathering machine	Solid metal lid, hinged metal lid, and a metal lid with a hole smaller than the defeathering machine's mouth, a customized metal lid with a hole and a pivot door (*2*,*3*).	To cover the defeathering machine during defeathering
Hot water	Maintained at 60°C	For scalding
Three buckets	Made of plastic	To store water inside booth at room temperature to use in the defeathering machine, to, t capture feathers and wastewater in front of the defeathering machine, and to store hot water for dipping the carcass outside the booth
Mug	Made of plastic; 2 L capacity	To pour water inside the defeathering machine
Electric socket	··	To turn on the defeathering machine

References

1. Berkeley Air Monitoring Group. Integrated cookstove assessment software (PICA) and the Particle and Temperature Sensor (PATS+) for air pollution measurements [cited 2025 Mar 28]. https://berkeleyair.com/wp-content/publications/Berkeley%20Air%20PICA%20and%20PATS+%20Flyer.pdf

2. Rimi NA, Fahad MH, Clark A, Sultana R, Hossain K, Saifullah MK, et al. Developing a method to estimate aerosol generation during poultry slaughtering and defeathering in Bangladesh: An experimental study. Build Environ. 2025;271:112621. PubMed https://doi.org/10.1016/j.buildenv.2025.112621

3. Wei J, Zhou J, Liu Y, Wu J, Jin T, Li Y, et al. A novel partial lid for mechanical defeatherers reduced aerosol dispersion during processing of avian influenza virus infected poultry. PLoS One. 2019;14:e0216478. PubMed https://doi.org/10.1371/journal.pone.0216478

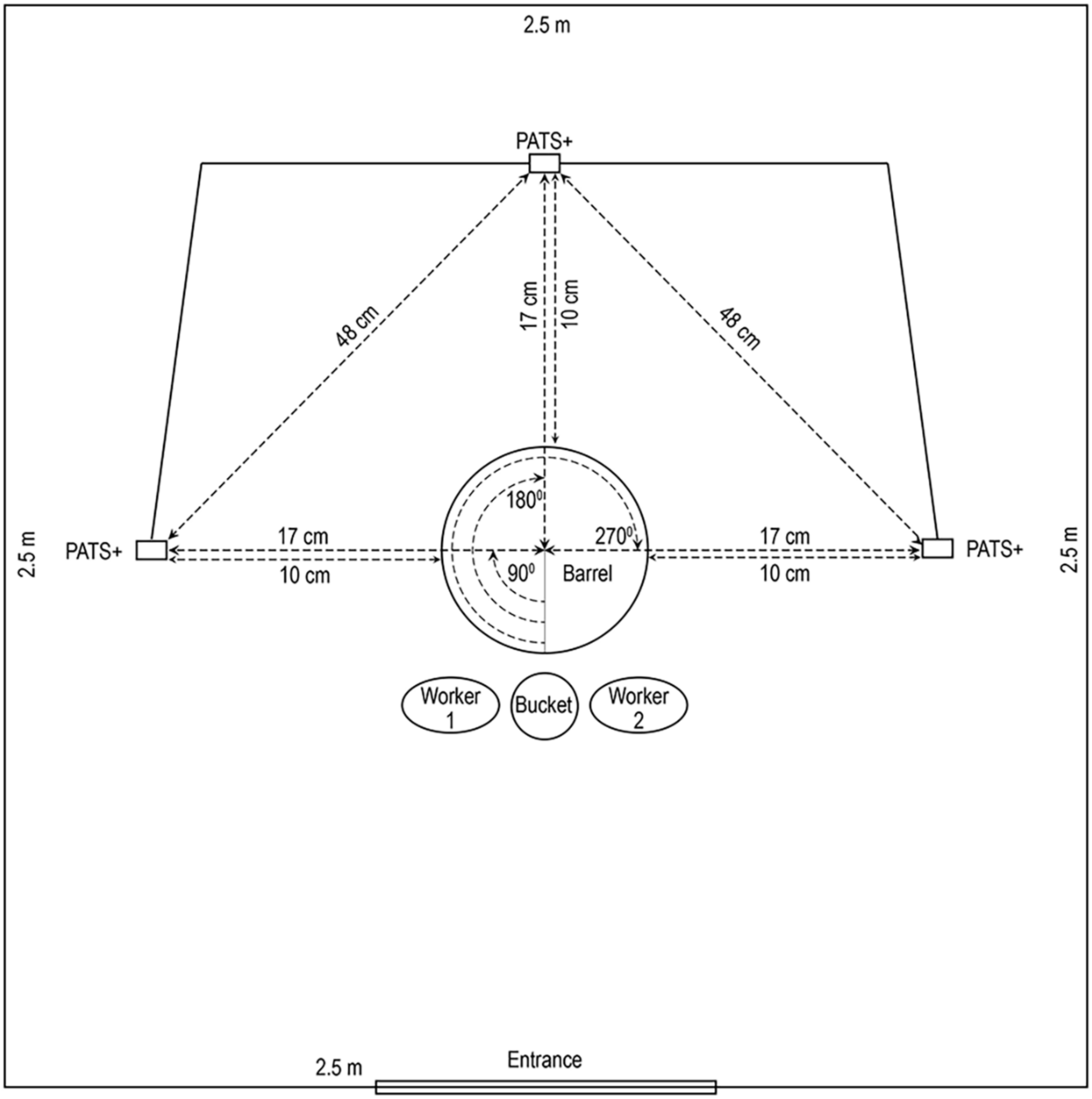

Appendix Figure 1. Slaughtering arrangements and particle monitors inside the booth for chicken slaughtering experiment at Bangladesh Livestock Research Institute (BLRI), Savar, Dhaka, 2020. A) Placement of particle monitor and slaughtering arrangement, B) open barrel without a lid, C) barrel covered with a solid lid, D) barrel covered with a star-cut lid, E) small cone, F) large cone.

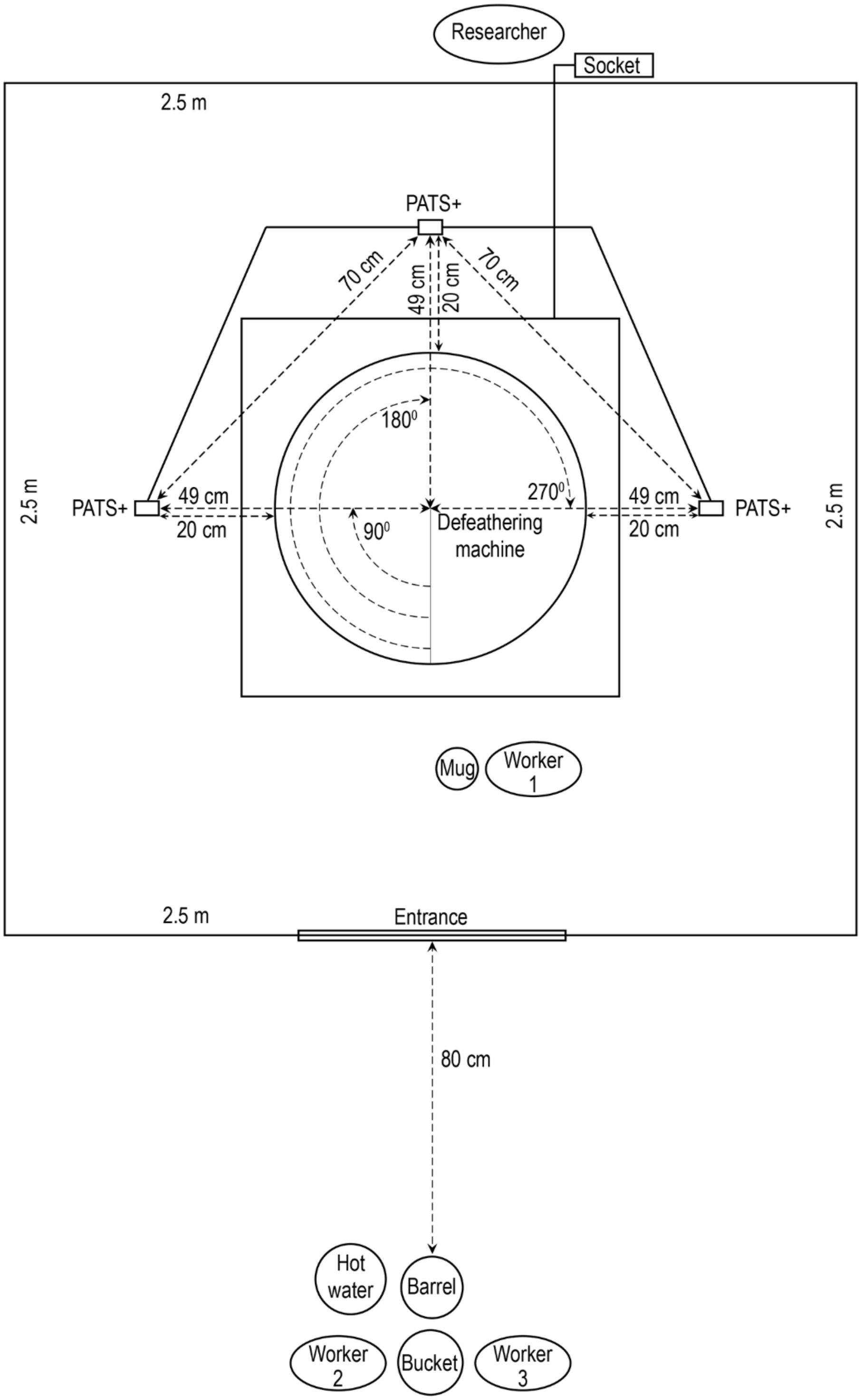

Appendix Figure 2. Defeathering arrangements and particle monitors inside the booth for chicken defeathering experiment at Bangladesh Livestock Research Institute (BLRI), Savar, Dhaka, 2020. A) Placement of particle monitor and defeathering arrangement, B) open machine without a lid, C) machine half-covered by a hinged lid, D) machine partially covered by a plate with a hole, E) machine fully covered by a lid with a hole and pivot door, F) machine fully covered by a slidable solid lid.
